# New Advances in Small Molecules Targeted at Viral Capsid–Genome Binding

**DOI:** 10.3390/ijms26146979

**Published:** 2025-07-20

**Authors:** Jiamei Li, Chengfeng Zhang, Benteng Li, Yuqing Wu

**Affiliations:** 1 State Key Laboratory for Supramolecular Structure and Materials, College of Chemistry, Jilin University, No. 2699 Qianjin Street, Changchun 130012, China; 2 Institute of Theoretical Chemistry, College of Chemistry, Jilin University, No. 2 Liutiao Road, Changchun 130023, China

**Keywords:** capsid protein (Cp), capsid–genome binding, antiviral strategy, small molecule, viral inhibitor

## Abstract

The capsid protein plays a crucial role in the viral life cycle. By interacting with the viral genome, it facilitates the assembly of the nucleocapsid, ultimately leading to the formation of the viral particle. Therefore, interfering with or disrupting the interaction between the capsid protein and viral genome can effectively inhibit viral replication and infection. This review focuses on elucidating the binding mechanisms between the capsid protein and the viral genome, as well as their potential applications as therapeutic targets. In particular, it summarizes the research progress on small-molecule drugs targeting the capsid–genome binding sites of dengue virus, HBV, and SARS-CoV-2. Notably, this review provides a detailed discussion on the mechanisms by which these small-molecule inhibitors interfere with the capsid–genome interaction, aiming to offer inspiration for the future development of novel antiviral drugs targeting the capsid–genome binding.

## 1. Introduction

The prevention and control of viral infections are currently encountering escalating challenges, particularly the emergence of drug-resistant strains and the rapid mutation of viral genomes [1,2]. There is an urgent imperative to develop innovative antiviral strategies that require a comprehensive understanding of both the viral structural biology and their functional mechanisms.

Viruses consist of three parts: a lipid bilayer containing glycoproteins, a nucleocapsid composed of coat proteins, and a viral genome [3]. The capsid plays a key role in genome encapsulation, which means that the coat protein must contain the ability to bind to the genome and thus ensure proper encapsulation of the viral genome. However, these coat proteins also appear to have specific roles; for example, in some flaviviruses, coat proteins can play a role in inhibiting interferon [4]. This means that it is important to understand the mechanism of the three interactions, protein–protein, protein–genome, and protein–small molecule substance, of the coat protein, whether it is to study possible antiviral sites on the coat protein or to circumvent the effect of the coat protein on antiviral tools. Among the various antiviral strategies, targeting the function of coat proteins in binding to the viral genome during the assembly process has demonstrated unique advantages in practical applications.

In recent years, preliminary research findings and theoretical models regarding the structures of various types of viral capsid protein (Cp) have been gradually published. However, due to the difficulty of molecular dynamics in handling complex biological macromolecular aggregation processes, the process by which viral Cp binds to the genome remained unclear at the time [5,6]. This led to a period of ambiguity in related inhibitor mechanism research. Currently, with the development of the structural biology techniques of mass spectrometry (ChIRP-MS) and cryogenic electron microscopy, in-depth research on Cp–genome interactions has yielded remarkable results [7]. These results are reflected in the determination of the structure and function of binding sites, both on Cp and on the genome. Also, the results discovered various molecules that affect Cp-binding genomes, including cell proteins, small DNA molecules, and medicinal small molecule inhibitors [8].

This review will focus on the critical roles of Cp in viral genome binding and assembly, highlighting their importance in the viral life cycle and potential as targets for antiviral strategies. Notably, this article will summarize research progress on capsid–genome inhibitors and their mechanisms of action for dengue virus (DENV), hepatitis B virus (HBV), and severe acute respiratory syndrome coronavirus 2 (SARS-CoV-2). By focusing on these areas, it aims to provide a comprehensive understanding of the role of Cp in genome biology and its potential as a target for antiviral interventions.

## 2. Structural Features of Cp–Genome Binding

### 2.1. General Structure and Virus Composition

The viral capsid is formed of repetitive Cps arranged closely together in a highly symmetrical manner, with the ability to inwardly bind the viral genome to outwardly dock membrane proteins. The coat proteins vary from virus to virus, and most coat proteins are driven by non-covalent interactions such as hydrogen bonding, hydrophobic forces, and ionic interactions. In general, 240 monomeric coat proteins are typically assembled into highly symmetric structures with T = 4, such as helices or icosahedral shapes [9], although an example composed of 180 Cp with T = 3 has also been mentioned in the literature [10]. The monomeric Cp forms dimers by dimerization to form the basic unit of Cp, and each monomer can be distinguished by amino acid residues into two structural domains, N-terminal and C-terminal [11]. It has been found that the N-terminal residues of Cp are mainly involved in binding to the viral genome, thus facilitating the formation of the viral core [3]. However, it has been shown that sites that interact with the genome are not necessarily at the N-terminus, such as in Getah virus, where assumed the N-terminal RNA interaction region is missing due to structural flexibility [12].

### 2.2. Genome-Binding Sites on Coat Proteins

The structural details of the Cp and its mode of binding to the genome vary among different viruses, making it difficult to establish a universal binding mechanism. However, with the accumulation of structural data for thousands of viral proteins across multiple viral families, four major capsid-based viral lineages can be identified, all of which share the icosahedral Cp structure [13]. They share similar capsid structures and binding mechanisms, so this section will briefly illustrate the Cp structure and binding mechanism using the widely studied flavivirus structure as an example.

Flaviviruses cause diseases in humans and animals through a wide range of mosquito transmissions [14]. The flavivirus Cp exhibits unique structural features that are critical for their function in viral assembly and genome packaging [15]. These features have been extensively studied through atomic-resolution structures obtained by nuclear magnetic resonance (NMR) spectroscopy and X-ray crystallography [16].

Zika virus (ZIKV), a member of the flavivirus family, was used as a demonstration as its dimer adopts a full α-helix conformation from the N- to the end of the C-terminus, denoted as α1, α2, α3, and α4 [6]. α4 is the largest helix in the structure, and its solvent-exposed region is rich in basic residues, so this is the presumed site of binding to RNA through electrostatic interactions. A continuous, positively charged region in coat proteins extends from the base of the molecule to the front and top of the homodimer, thus forming an uninterrupted pathway that wraps around the entire molecule. A study testing ssDNA binding in multiple mutants by electrophoretic mobility shift assay (EMSA) showed that this positively charged pathway plays a key role in binding genomic functions [17]. The results show that the positively charged pathway that wraps the ZIKV capsid molecule plays a key role in nucleic acid binding. This feature has also been found in other virus structures [18]. Most Cp have a highly electropositive surface, crucial for binding to the negatively charged viral RNA genome. This feature is maintained by contacts between protein subunits, ensuring the stability of the capsid structure [19].

Another notable feature of Cp is the presence of a flexible helix (α). This structural flexibility is thought to regulate molecular recognition and interactions with the viral RNA genome. The flexibility of α allows the capsid protein to adapt to different RNA structures during packaging [20]. In addition, the unique three-dimensional structure of Cp is also an important factor in the binding of Cp to the genome. Cp forms intertwined homodimers, pentamers, or hexamers in solution, which are essential for the assembly of the viral nucleocapsid. Such oligomerization is driven by specific interactions between the helices and loops of the protein, providing a scaffold for RNA binding and packaging [21]. The atomic-resolution structures reveal a unique fold that is characterized by a predominantly helical structure with a central core and flexible loops. The unique arrangement of these structural elements is essential for the stability and function of the capsid [22].

### 2.3. Protein-Binding Sites on Genome

The binding is bidirectional, meaning that the corresponding binding sites on the genome are equally important for the Cp binding process. A recent study highlights human rhinovirus (RV) as the first of its kind among small RNA viruses and human viruses because of the known organization of the viral ssRNA genome and its interaction with the capsid. The assembly process is mediated by sequence/structural elements in genomic RNA (called packaging signals, PS) [23]. PS can participate in the packaging process by binding to specific sites on the surface of the CP subunit, a process that has also been observed in many other viruses [24]. High-resolution cryo-electron micrographs of viral particles (strain RV-B14) with well-defined electron densities allow for the direct visualization of 30 double-stranded (ds) RNA elements [23], each folded in a canonical A form. The elements contain 13 nucleotide pairs flanked on each end by an unpaired purine nucleotide symmetrically bound to the concave surface of the inner shell wall surrounding the 2-fold axis of each coat. Thirty RNA double-stranded body elements define the edge of the lower wall of the dodecahedral cage of the coat. The flanking purine and sugar-phosphate backbones of the double-stranded body establish symmetric interactions with specific coat residues, including two symmetry-associated Trp residues, a positively charged side chain that tightly surrounds each RNA double-stranded body, and residues involved in the formation of hydrogen bonds with the sugar-phosphate backbone [7]. Research in this direction may lead to the development of novel antiviral strategies that inhibit viral encapsulation by targeting genomic binding sites in the future.

## 3. Small Molecules Targeting Capsid Protein–Genome Binding

### 3.1. Small Molecules Disrupting Capsid Subunit Interactions

Small molecules have emerged as a promising strategy for disrupting Cp–genome binding, thereby inhibiting viral assembly and replication. Core methods include targeting specific functional sites of Cp, competing with Cp binding, and interfering with the normal structure of Cp. Methods such as binding free energy calculations using the MM/GBSA method, molecular dynamics simulation analysis, etc., can determine the stability of potential drugs and the effect of binding to coat protein [25,26]. Recent relevant antiviral research findings will be summarized below by virus type.

### 3.2. Small-Molecule Inhibitors for Dengue Viruses

**Figure 1:**
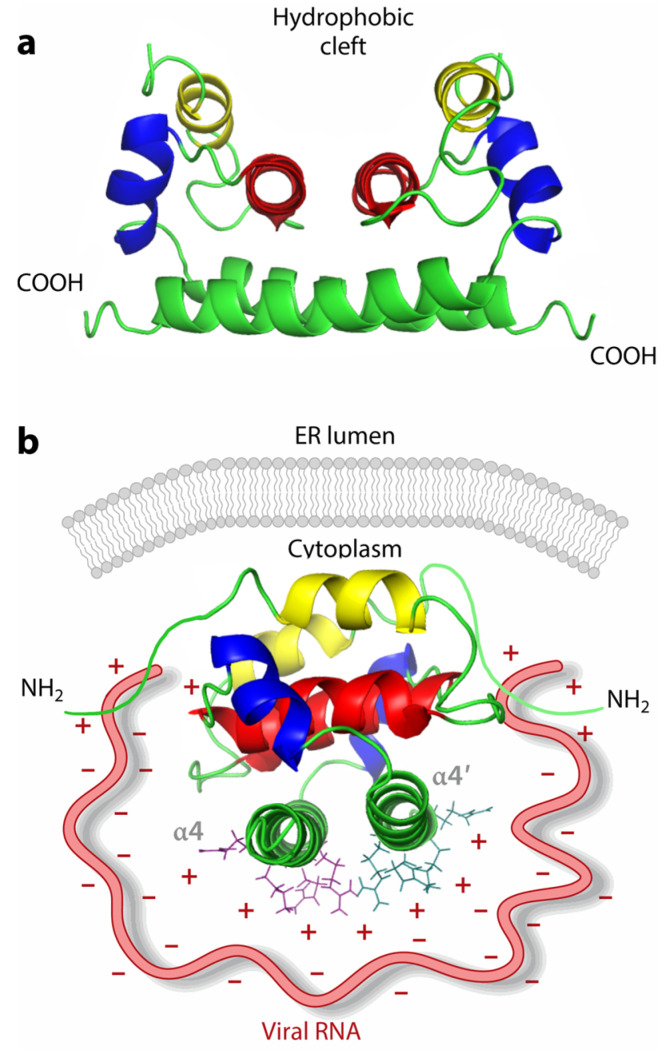
(**a**) Structure of the dengue virus (DENV) capsid protein; α1 in yellow, α2 in red, α3 in blue, and α4 in green. (**b**) Positive charge distribution at the C-terminal side (α4-α4′interface) for viral RNA binding. Reproduced from ref. [28].

Dengue virus (DENV), a significant member of the flavivirus family, causes viral hemorrhagic fever and has emerged as a major global health concern. DENV Cp is a 12 kDa highly basic protein containing 100 amino acid residues that form a homodimer [27]. Structural studies showed that the monomer has four α-helices (α1-α4), and the dimer exhibits an asymmetric charge distribution, with the accumulation of basic residues on one side and a concave, nonpolar surface on the other (Figure 1).

The C-terminal positively charged region (α4-α4′ interface) of Cp serves as a critical functional domain that binds viral RNA during genome packaging (Figure 1b). Defects in it result in the formation of empty virus-like particles (VLPs) that contain no viral genome [28]. It is a very promising antiviral site. However, early research efforts were hampered by limited structural understanding of Cp-RNA interactions and incomplete knowledge of the viral life cycle. But the frequency of relevant research results in recent years has raised hopes for understanding past research results and finding novel Cp-RNA binding-targeting inhibitors, potentially ushering in a new era of transformative dengue therapeutics.

In 2024, Panday et al. made significant progress in identifying potential inhibitors of DENV Cp by screening Food and Drug Administration-approved drugs through computer simulation techniques [29]. These compounds specifically target the α1-helix domain of the viral Cp. Among them, Nordihydroguaiaretic acid (NA) exhibited excellent binding affinity, with a binding energy of −11.66 kcal/mol to DENV Cp, while Ifenprodil tartrate (IT), Lathyrol (LT), and Safinamide Mesylate (SM) also showed promising inhibitory potential. Molecular docking analysis revealed that NA could form stable hydrogen bonds with the key residue Phe33 in chains A and B, occupy the hydrophobic pocket of the capsid protein, and interact via hydrophobic forces with residues such as Leu29, Leu35, and Met37. This specific binding induces conformational changes in the α1-helix. Although the inhibitor does not directly compete with the RNA-binding site located in the α4-helix, it may affect its spatial orientation and dynamic properties, indirectly interfering with the RNA-binding function of Cp, thereby blocking viral genome packaging.

However, the authors do not give a detailed answer when it comes to delving into the question of why interaction with residues such as those above can hinder Cp-RNA binding. The surprise was that the potential small molecule inhibitor identified by Panday et al. binds to Cp at the α1 helix A-strand B-strand, whereas, as early as 2004, Ma et al. proposed that of the four helices, it is the α4 helix that binds to RNA through electrostatic interactions [30]. That is, the potential inhibitors identified by Panday et al. do not inhibit Cp–genome binding by competing with RNA for the binding site. Therefore, this study provides an important theoretical basis for developing novel anti-DENV drugs. The α1-helix of the capsid protein can serve as an allosteric target for anti-DENV drugs, avoiding direct targeting of the RNA-binding region, which may reduce the risk of viral resistance. However, follow-up studies combining in vitro and in vivo viral inhibition experiments are needed to further validate its actual antiviral activity and elucidate the specific molecular mechanism by which Cp conformational changes lead to impaired RNA packaging.

At the same time, novel results have been obtained for studies related to the key residues on α4 that bind the genome. In 2022, Mebus-Antunes et al. experimentally analyzed RNAs of different sequences interacting with Cp in the same way, concluding that Cp interacts with RNA in a sequence-independent manner [31]. In 2023, Neves-Martins et al. made significant progress on studies related to key residues for Cp-RNA binding [28]. They are based on the fact that the in vitro assembly of DENV nuclear capsid-like particles (NCLP) requires coordinated neutralization of the positive charge of Cp by interaction with size-specific nucleic acids or negatively charged surfaces [15]. In conjunction with structural analysis of positively charged DENVC surfaces, it was hypothesized and verified that the high positive site conferred by arginine 85 (R85) and lysine 86 (K86) residues in the α4 helix would be the first point to be neutralized by RNA binding, thereby triggering NCLP assembly. Then, they designed a mutant in which DENVC R85 was replaced by a cysteine residue (R85C mutant), resulting in the loss of the α4/α4′-positive site. The experimental results showed successful self-assembly of DENVC R85C into clathrate-like particles (CLPs) in solution in the absence of nucleic acids, confirming the hypothesis that this is the first study to establish efficient in vitro self-assembly of DENVC in solution in the absence of nucleic acids, revealing that the highly positive site conferred by arginine 85 (R85) and lysine 86 (K86) residues would be the point of binding to and neutralization of RNA, thereby triggering Nucleic acid (NDA) binding. This also provides a novel direction: the residues on the α4 helix that provide the highly positive site are essential for Cp-RNA binding, and their absence leads to the creation of an empty nucleosome.

Recently, Chaudhuri et al. further confirmed through molecular dynamics simulations that the positively charged residues (R85/K86) on the α4 helix are key sites for RNA binding [32]. Additionally, studies have shown that after the positive charge of the α4 helix is neutralized by RNA, the conformation of Cp changes, thereby promoting Cp self-assembly, which is highly consistent with the experimental results of Neves-Martins et al. These findings further deepen our understanding of the interaction between DENV Cp and RNA. At the same time, the identification of these key residues provides an important basis for developing novel antiviral strategies against DENV.

However, the development of effective inhibitors remains challenging. Expanding research to include structural and functional analyses under physiological conditions could reveal inhibitor stability and efficacy dynamics. Additionally, investigating long-term effects and resistance in DENV serotypes may enhance treatment viability. This multidisciplinary approach addresses dengue’s global health threat and advances antiviral drug development.

### 3.3. Example of CpAMs Targeting on Hepatitis B Virus

#### 3.3.1. Hepatitis B Virus Nucleocapsid Assembly

Hepatitis B virus (HBV) is a DNA virus of the Hepadnaviridae family, in which the pregenomic RNA (pgRNA) and viral polymerase (Pol) are selectively packaged into an assembled capsid formed by the viral Cp. In the past, the assembly principles were difficult to specify due to the lack of a cell-free system capable of reconfiguring selective HBV Pol-pgRNA packaging into a fully assembled capsid [33]; however, it has been resolved recently.

**Figure 2:**
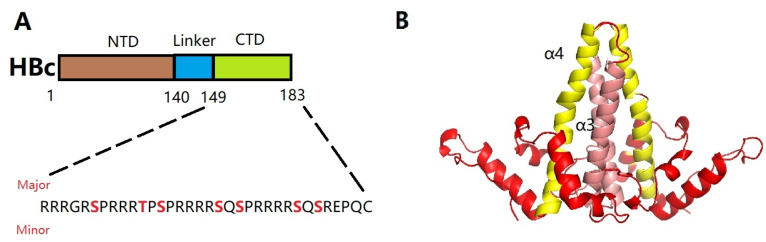
(**A**) Schematic diagram of HBc (genotype D), with each domain labeled at the top and amino acid positions labeled with numbers at the bottom. The CTD sequence is shown, with serine and threonine residues in the CTD that are targets for phosphorylation highlighted, including three major phosphorylation sites and four minor sites (bold red). (**B**) The HBc dimer (PDB:1QGT) is primarily composed of α-helices. The dimer interface is formed by a quadruple helix bundle composed of α1 (red), α2 (red), α3 (pink) and α4 (yellow) helices from each monomer.

The Cp formed by HBV consists of an N-terminal structural domain (NTD, residues 1–140) and an arginine-rich C-terminal structural domain (CTD, residues 150–183), which are linked by a short splice peptide (residues 141–149). This junction region is not a simple spacer region between the NTD and CTD, but a functional site for capsid assembly, pgRNA packaging, and DNA synthesis [34]. Therefore, it is also a target site for antiviral strategies. In addition, the coat protein of hepatitis B virus (HBc) has three major serine–proline phosphorylation motifs and four minor serine or threonine phosphorylation sites that are phosphorylated with high frequency in its CTD 104, 105, and 106 (Figure 2A) [35]. These sites are dynamically phosphorylated and dephosphorylated and are key regulators of genome packaging. Phosphorylation of the HBc CTD reduces the positive charge of the arginine-rich CTD, thereby reducing the binding RNA capacity and preventing nonspecific RNA packaging [36]. Whereas dephosphorylation occurs during HBV pgRNA packaging, dephosphorylating HBc in the CTD at the Ser 170 site promotes selective Pol-pgRNA packaging [37].

#### 3.3.2. A Brief Introduction to Research on CpAM

While current therapies for chronic hepatitis B (CHB), including nucleoside analogs (NAs) and PEGylated-interferon-alpha (PEG-IFN-alpha), have been deeply investigated. However, they are rarely curative for chronic hepatitis B (CHB) due to the inability to eliminate viral covalently closed circular DNA (cccDNA) [38]. This limitation has driven the urgent need for novel antiviral strategies. Therefore, new antiviral drugs are urgently needed. It has been found that deviations in Cp-genome assembly not only lead to the formation of abnormal virus particles that are not infectious, but also affect the establishment and replenishment of cccDNA libraries, leading to cccDNA depletion and, finally, HBV clearance [39]. Compounds with such functions are named core protein alteration modifiers (CpAMs) [40].

**Figure 3:**
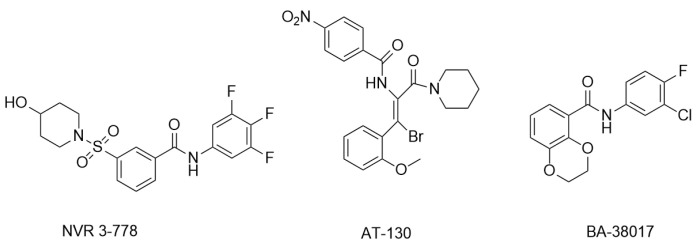
Representative compounds of type II CpAM.

With the intensive research on CpAMs’ inhibition of HBV replication, CpAMs have been classified into two major types. Type I, represented by heteroaryldihydropyrimidines (HAPs), induces aberrant Cp assembly and disrupts capsid assembly by binding Cp at the dimer-dimer interface pocket, resulting in Cp degradation. Type II, represented by phenylpropionamide (PPA), prevents the assembly of nucleocapsids containing pgRNA, resulting in defective virus particles with empty capsids. Both types act at the Cp dimer-dimer interface site. Of note, despite the proven anti-HBV capacity of type II CpAM as early as 2019, the action mechanism of type II remained unclear until recent years, when it was intensively studied to gain tremendous progress [41]. In this review, only studies related to type II CpAM hindering Cp-genome binding are discussed. The main representatives are sulfonamide inhibitors (NVR 3-778), acrylamide inhibitors (AT-130), and benzamide inhibitors (BA-38017) (Figure 3).

#### 3.3.3. Sulfamoylbenzamide and Its Derivatives

Sulfamoylbenzamide (SBA) was identified as a new chemotype of CpAM from a high-throughput screen. In 2013, Campagna et al. experimentally determined that SBA derivatives inhibit the assembly of HBV nucleocapsids. The experiments were performed using a high-throughput screening assay to determine the amount of HBV DNA in cell lysates. A total of 40 compounds reduced the amount of HBV core DNA by more than 70%, of which 36 compounds shared a sulfonamide–benzamide pharmacophore with functional groups at the sulfonamide, amide, and intermediate phenyl sites.

**Figure 4:**
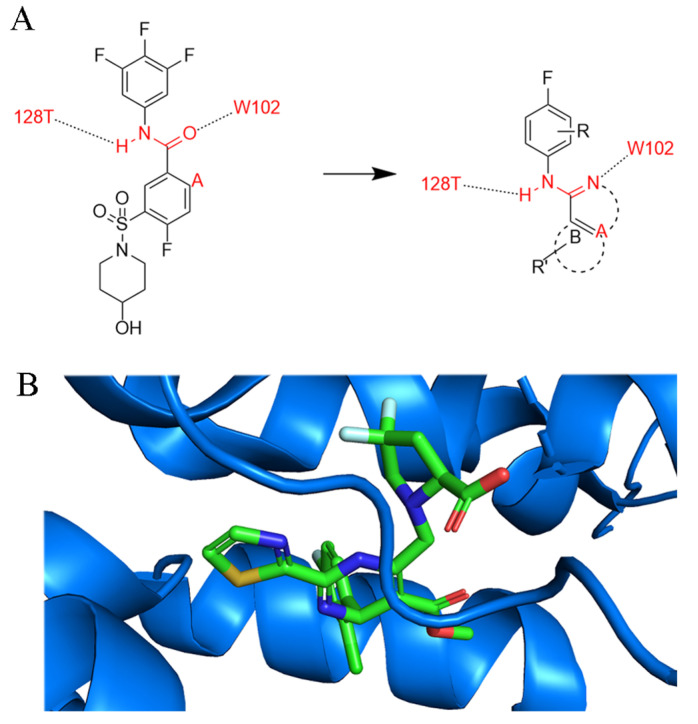
Design of novel CAMs through conformation constraint. (**A**) Rational design of CpAM (**B**). Merging of HAP-R01 (PDB: 5WRE) in the binding site. Different colors represent various elements of the HAP-R01 molecule. Blue represents Cp.

In 2020, Wang et al. described the discovery of (1H-indazol-5-yl)sulfonamide and (1H-pyrazolo[3,4-c]pyridin-5-yl)sulfonamide as new chemotypes of CAM with lower cytotoxicity and higher potency by restricting the conformation of SBA [42]. Figure 4A shows their design of novel inhibitors, with consideration of binding sites in Figure 4B. In this regard, compounds with indimidazole scaffolds were synthesized to inhibit extracellular HBV DNA in HepAD38 cells with an *EC*_50_ value of 1.28 μM, and the 2-N atoms on the indimidazole scaffolds were able to form a suitable hydrogen bond with *W102* of the HBV core protein. Moreover, the 1-N atoms provide an additional site for structural optimization, which is what distinguishes indimidazoles from other scaffolds.

#### 3.3.4. Other Newly Developed Type-II CpAM

In 2022, Liu et al. designed three series of compounds using the classical drug design strategy of pharmacophore hybridization, bioequivalence, and scaffold jumping using NVR 3-778 and BA-38017 as lead compounds and validated their antiviral effect [43]. Among them, series I showed promising antiviral ability and inhibition of capsid assembly potency. In series I, it was found that the introduction of a non-polar hydrophobic group on R1, similar to a five-membered ring, resulted in better anti-HBV DNA replication activity and lower cytotoxicity. One of the compounds showing the most potent anti-HBV DNA replication activity reached an *EC*_50_ = 0.50 ± 0.07 μM, which was superior to compound BA-38017 (*EC*_50_ = 1.94 ± 0.19 μM, CC_50_ > 100 μM). The *IC*_50_ value was 4.22 μM, which was almost the same as the lead compounds NVR 3-778 (*IC*_50_ = 1.25 μM) and BA-38017 (*IC*_50_ = 1.70 μM).

In 2024, Cole et al. provided new ideas in the search for a novel type-II CpAM. The new CpAM forms a distinct anchored hydrogen bond primarily with the *W102* side chain and provides a hydrogen bond donor [44]. It also interacts with the accessible *T128* receptor provided by the side chain oxygen and contains the fragments bound in the hydrophobic pocket mentioned above. Between these structural cores with hydrogen bond acceptor and/or donor capabilities, a spacer needs to be incorporated that can take advantage of the proximity of residues *L140*, *S121*, and *S141* and provide the suggested spatial site-blocking projections.

Based on this assumption, they envisioned that the primary consideration of hydrophobic elements could be achieved by substituting phenylurea. The methylene spacer in the core would allow exploration of the binding pocket and possibly the installation of a second urea nitrogen by reductive amination chemistry using a substituted aldehyde. And the bicyclic core also provides the suggested spatial site-blocking projections that occupy the three-dimensional space demonstrated in the structural biology results. This has led to the design of the clinical candidate AB-836, which is currently undergoing phase 1 clinical studies [45].

**Figure 5:**
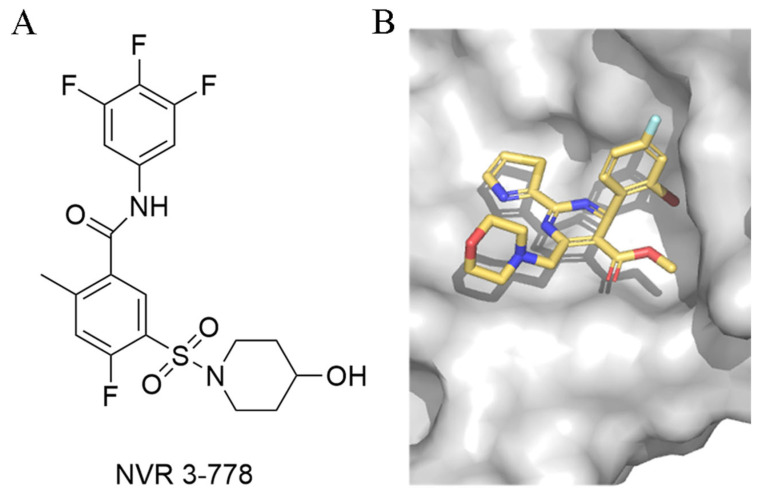
(**A**). NVR 3-778 (**B**). NVR 3-778 binds to chain B of HBV Cp (PDB code: 5E0I).

Moreover, in 2025, Wang et al. identified two key binding hotspots on HBV Cp by systematically investigating the binding mechanism and exploring structure–activity relationship (SAR) optimization strategies [43]. The 2-cyclopropyl-thiourea benzamide (CP-TBA) derivatives have been shown to be novel and effective CpAMs through structurally diverse modifications and new binding site occupancy strategies. Their starting point came from exploring the structural reasons for the difference in activity between NVR 3-778 (Figure 5) and GLS4, two known CpAMs (the former is more than 10 times lower than the latter), and systematically exploring SAR optimization strategies. Comparative structural analysis reveals conformational congruence between NVR 3-778 and GLS4 within the Cp-binding pocket, but exposes key differences in hydrophobic interactions [46]. As a result, they introduced hydrophobic substituents, leading to the development of the Ia and Ib series. At the same time, the solvent-exposed sulfonamide portion of NVR 3-778 provided an opportunity for structural diversification. Using a strategy of bioequivalence and structural modification, they introduced various connectors such as aminoacyl and thiourea groups into this region.

This design strategy was subsequently validated by biological evaluation of the target compounds. The introduction of cyclopropyl groups in the tolerance region was shown to enhance the overall activity of the compounds. Moreover, the thiourea moiety in the solvent-exposed region proved to be favorable for active expression. Finally, this work disclosed the representative compound **17e**, which achieved enhanced anti-HBV activity in HepAD38 cells (*EC*_50_ = 0.033 μM) over the lead compound NVR 3–778 (*EC*_50_ = 0.35 μM) and slightly less than GLS4 (*EC*_50_ = 0.022 μM). Moreover, **17e** illustrated remarkable potency in the HBV-infected cell line HLCZ01, with an *EC*_50_ value of 0.012 μM. MD simulations illustrated hydrogen bonds of it with key residues Trp102, Thr128, and Leu140, leading to commendable metabolic stability in human plasma, but it was more rapidly metabolized in human liver microsomes. Additionally, **17e** manifested with a suboptimal profile, with a bioavailability of merely 28.9% at a dosage of 5 mg/kg. Moreover, treatment with **17e** for 20 days demonstrated potent anti-HBV activity in the HBV carrier mice model.

### 3.4. Small-Molecule Inhibitors for SARS-CoV-2

Severe acute respiratory syndrome coronavirus 2 (SARS-CoV-2) belongs to the β-coronavirus family, which caused the COVID-19 pandemic. Coronavirus nucleocapsid protein (N) exhibits a highly conserved structure in which the N-terminal structural domain (NTD) and C-terminal structural domain (CTD) are sandwiched by three intrinsically disordered regions (IDRs) known as the N-arm, the central junction region (LKR), and the C-tail. Both NTD and CTD can bind to RNA, and CTD also functions in protein dimerization and interacts with viral packaging signals [47,48]. Therefore, targeting NTDs and CTDs to interfere with RNA encapsidation and inhibit viral replication is an effective antiviral strategy for SARS-CoV-2 [49].

Wang et al. designed a series of phenanthridine derivatives based on the NTD of the SARS-CoV-2 nucleocapsid protein [50]. Among them, compound **12** (*EC_50_* = 3.69 μM) and compound **16** (*EC_50_* = 2.18 μM) exhibited significant antiviral activity and low cytotoxicity (CC_50_ > 200 μM). SPR experiments confirmed that compounds **12** and **16** tightly bind to the SARS-CoV-2 N protein, both with a *K*_D_ of 7.82 μM. Molecular docking of compounds **12** and **16** bound to the NTD crystal structure indicates the presence of three predicted pockets. Notably, in pocket A, the methoxy and amino groups of compound **12** form hydrogen bonds with Tyr109 and Pro151, while the amide and fluorine atoms of compound **16** establish hydrogen bonds with Tyr109 and Arg149, respectively. The phenanthridine core of both compounds also engages in π-cation or π-anion interactions with residues such as Arg107, Arg149, and Glu174, further stabilizing the binding. Additionally, site-directed mutagenesis experiments confirmed that Tyr109 is a critical residue for the binding of these compounds to N-NTD.

Since N-NTD plays a key role in viral RNA binding and replication, compounds **12** and **16** may interfere with the interaction between the N protein and the viral RNA genome by binding to NTD, thereby inhibiting viral genome packaging and replication. This mechanism provides a new direction for the development of novel anti-SARS-CoV-2 drugs, and also suggests that N-NTD may be a potential broad-spectrum target for anti-coronavirus therapeutics.

With in-depth research into Cp-binding genomic processes, some brand-new discoveries have emerged, such as the phenomenon of liquid–liquid phase separation that occurs during the process. Liquid–liquid phase separation (LLPS) of viruses refers to the phenomenon in which Cp and genome condense into aggregates through multivalent interactions, forming semi-liquid and semi-solid compartments distinct from the surrounding fluid [51]. Viruses utilize LLPS during their replication cycle to generate membrane-free organelles from nucleic acids and proteins, isolating them from other cellular components. LLPS is an important mechanism of SARS-CoV-2 NPs involved in viral assembly, replication, and immune regulation, which has become a new and promising research direction and has made great progress recently.

In 2021, Wang et al. determined that the Cp-RNA polymer of SARS-CoV-2 is produced by the RNA-induced Cp LLPS process and found that (−)-gallocatechin gallate (GCG) can inhibit its LLPS process and thus inhibit viral replication [52]. After identifying this novel possibility of inhibiting viral encapsidation, they tested inhibitors known to have an inhibitory effect but with uncertain mechanisms and found that GCG can block RNA-triggered LLPS of Cp.

In 2023, Dang et al. demonstrated that nucleic acids regulate LLPS through dynamic and multivalent interactions of folded NTD/CTD and Arg/Lys residues within the intrinsically disordered regions (IDRs), revealing a high-resolution mechanism of LLPS [53]. Also, they found that ATP and nucleic acids competitively modulate LLPS of the SARS-CoV-2 nucleocapsid protein, revealing ATP as a class of small molecules able to target LLPS. A 32-oligomer stem-loop II nucleic acid motif S2m derived from SARS-CoV-2 gRNA was used, which was shown to induce and solubilize LLPS mainly through specific binding to folded NTD and Arg/Lys residues in the IDR of the Cp disordered region. Therefore, a hypothetical model is proposed that N (1–249) and S2m can dynamically and multivalently interact with each other on NTD and IDR Arg/Lys residues to form large dynamic cross-linking complexes that behave as liquid droplets.

This line of research now provides a novel perspective on the molecular barriers to Cp–genome binding. This phenomenon has also been observed in other viral systems involving proteins such as FUS and TDP-43. For instance, RNA and single-stranded DNA (ssDNA) have been demonstrated to biphasically modulate the liquid–liquid phase separation (LLPS) of FUS and TDP-43, respectively [54,55]. This emerging direction not only facilitates a more comprehensive understanding of the mechanisms underlying Cp encapsulation and the inhibitor action, but also opens up new avenues for exploring these processes. Furthermore, this approach holds potential for the development of innovative antiviral therapeutic strategies.

### 3.5. Summary on the Discovery of Potential Small Molecules

Overall, the search for new potential small-molecule inhibitors is based on renewed in-depth studies of the mechanisms and sites of action of existing inhibitors. Viral systems should be selected based on Structural tractability (availability of high-resolution Cp-genome complex structures) and Experimental tractability, for instance, established biochemical assays for assembly. The binding site is then analyzed by computational methods such as molecular dynamics simulations (AMBER, GROMACS). And protein–RNA docking (HADDOCK) is used to predict key contact residues and simulate the binding. The computational results will be validated by experiments such as cryo-electron microscopy and mutagenesis of essential residue clusters in the Cp RNA binding domain. On this basis, a targeted search for potential small molecules can be carried out. The potential inhibitors can be screened from methods such as searching for drugs with specific structures through known drug libraries, or the targeted modification of small molecules with known structural functions. The screened candidates can then be further experimentally validated for their efficacy. This is a generic search for potential small-molecule inhibitors. It is believed that more methods and strategies will be revealed in future studies.

## 4. Conclusions and Perspectives

This paper aims to provide a systematic overview and summary of antiviral strategies targeting Cp–genome binding. First, it summarizes the structural mechanisms of the binding process, covering how the Cp structure binds to the genome through a three-dimensional specific structure, and appropriately expands on research related to genome binding sites. Then, this paper focuses on different viruses, analyzing the representative small-molecule inhibitors based on specific structural characteristics, and attempts to combine the latest research results with the unclear mechanisms of past inhibitors. It aims to serve as a bridge connecting past and present, as past technical limitations led to unclear antiviral mechanisms, while recent years’ new research methods have provided deeper structural details, offering the possibility to refine and improve related inhibitors. It is hoped that the novel and promising antiviral strategies summarized in this paper will pave the way for new antiviral drug development pathways to address increasingly severe viral challenges. We believe that this antiviral strategy will be applied more widely, and that future research will yield clearer mechanisms of interference and more specific microscopic sites of action for inhibitors. Additionally, inhibitors that act on the viral icosahedral cage structure or mediate LLPS processes may also be the subject of further research.

However, the lack of high-resolution techniques for tracking these in vivo interactions in real time has limited insights into mechanisms. Also, future research should prioritize (1) advanced structural biology methods (e.g., cryo-electron microscopy and time-resolved crystallography) to capture transient binding states, (2) computational modeling to predict hotspots of interactions across viral families, and (3) innovative drug-delivery strategies to target the capsid–genome interface without extra-host effects. Bridging these gaps could lead to the development of novel antiviral drugs and inspire RNA/DNA-targeted therapies beyond virology, such as gene delivery systems.

## Data Availability

Data are contained within the article.
